# Independent causal effect of migraines on Alzheimer’s disease risk: a multivariate Mendelian randomization study

**DOI:** 10.3389/fneur.2024.1401880

**Published:** 2024-06-05

**Authors:** Chengfeng Xu, Wen Wu, Yuchao Fan, Shuying Zhu

**Affiliations:** ^1^Department of Anesthesiology, Sichuan Clinical Research Center for Cancer, Sichuan Cancer Hospital and Institute, Sichuan Cancer Center, Affiliated Cancer Hospital of University of Electronic Science and Technology of China, Chengdu, China; ^2^Department of Anesthesiology, Xichang People's Hospital, Xichang, Sichuan, China

**Keywords:** migraine, Alzheimer’s disease, univariable Mendelian randomization, multivariable Mendelian randomization, meta-analysis, causal effect

## Abstract

**Background:**

The observational studies investigated the impact of migraine on Alzheimer’s Disease (AD). However, these findings were limited by confounding factors and reverse causation, leading to contradictory results.

**Methods:**

We utilized Univariable Mendelian Randomization (UVMR) to explore the link between migraine (13,971 cases/470,627 controls) and AD risk (Bellenguez et al., 39,106 cases/46,828 controls; FinnGen, 111,471 cases/111,471 controls). Meta-analysis was performed for comprehensive synthesis. Employing Multivariable Mendelian Randomization (MVMR), we created models incorporating migraine and 35 potential AD risk factors, examining migraine’s independent impact on AD onset risk under considering these factors.

**Results:**

The meta-analysis of inverse variance weighted MR results, combining data from Bellenguez et al. (odds ratio (OR) [95% confidence interval (CI)]: 1.5717 [1.1868–2.0814], p = 0.0016) and FinnGen (OR [95% CI]: 1.2904 [0.5419–3.0730], *p* = 0.5646), provided evidence for a causal relationship between genetically predicted migraine and the heightened risk of AD occurrence (OR [95% CI]: 1.54 [1.18, 2.00], *p* < 0.01). After adjusting for Diastolic blood pressure (OR [95% CI]: 1.4120 [0.8487–2.3493], *p* = 0.1840) and Tumor necrosis factor alpha (OR [95% CI]: 1.2411 [0.8352–1.8443], *p* = 0.2852), no discernible association was detected between migraine and the risk of AD.

**Conclusion:**

This study offers compelling evidence indicating a significant correlation between genetically predicted migraine and an elevated risk of AD.

## Introduction

Alzheimer’s disease (AD) and migraine are ranked as the second and third most burdensome neurological disorders in the United States, respectively (1). Current estimates indicate that over 50 million individuals worldwide are affected by AD, incurring annual expenditures surpassing one trillion US dollars (2). This figure is expected to rise significantly in the upcoming decades due to the growing global aging population (3). The impact of AD extends beyond individual patients, profoundly affecting families and society as a whole. In the initial stages, patients may display symptoms such as diminished memory, slowed cognition, and emotional instability, gradually compromising their ability to live independently (3). With disease progression, patients may lose the ability to recognize their own family members, resulting in considerable emotional distress and anguish among their loved ones (4). Moreover, individuals with AD often require extended caregiving, imposing significant financial strain on families and subjecting caregivers to exhaustive physical and emotional challenges (4). Furthermore, the socioeconomic landscape is significantly impacted, including increased pressure on healthcare resources, a decrease in the labor force, and a heightened burden on social welfare systems. The rising economic costs associated with long-term care, medical expenses, and loss of productivity present ongoing challenges to national finances (5).

Migraine, a prevalent neurological disorder, is a significant global health concern. According to a systematic analysis conducted in the 2016 Global Burden of Disease study, approximately 1 billion people worldwide suffer from migraine annually (6). Remarkably, migraines rank as the second leading cause of disability-adjusted life years across all age groups globally (7). While commonly afflicting young to middle-aged individuals, migraines can affect both children and the elderly. Characterized by intense headache episodes, migraines are often accompanied by symptoms such as nausea, vomiting, and heightened sensitivity to light and sound (8). Typically lasting between 4 to 72 h, the severity of migraines varies (9, 10), impairing patients’ ability to function normally in work and daily life (11, 12). Recent studies have explored potential links between migraines and AD. However, these investigations have yielded conflicting conclusions (13–18), possibly attributed to differences in research methods, sample sizes, and study designs (19, 20).

Research exploring the association between migraine and AD holds profound scientific and clinical significance. Investigating the link between these conditions offers the potential for novel insights into treatment strategies. Moreover, the investigation of the connection between migraine and AD has the potential to advance cognitive health in public health and clinical practice. Once the link is established, healthcare institutions and public health authorities can enhance health education for migraine sufferers and their families, increasing awareness of the risk of AD. Furthermore, clinical practitioners diagnosing and treating migraine patients can become more vigilant regarding the potential risk of AD, enabling early intervention and management. This proactive approach aids in the early identification of individuals at risk for AD, enabling the implementation of appropriate prevention and intervention measures. This bears significance in the endeavor to mitigate the development of AD and alleviate its socio-economic burden (19, 20).

Nevertheless, it is important to highlight that existing investigations concerning the link between migraines and AD predominantly comprise retrospective studies (13–18), thereby introducing complexities in ascertaining causality. Rigorous randomized controlled trials (RCTs) grapple with practical challenges within this realm, encompassing concerns related to population bias, disparities in measuring and reporting outcomes, intricacies associated with prolonged follow-up durations, and the presence of diverse confounding elements (13–16, 19, 20). Moreover, addressing the potential for reverse causation in study design proves to be notably intricate t (13). Recognizing the inherent constraints of retrospective studies in establishing causal connections, it becomes essential to employ alternative research methodologies to alleviate the influence of confounding variables.

In recent times, the Mendelian randomization (MR) approach has surfaced as a novel and promising tool within the realm of epidemiology, primarily employed for the examination of genetic causal associations between intricate risk factors and diseases (21). In contrast to conventional observational investigations, MR analysis offers unique advantages, as it leverages genetic variation as a means to scrutinize the causal links connecting exposure variables and resultant outcomes. Given that genetic variations manifest random distribution during gamete formation and often remain unaffected by environmental and lifestyle influences, MR serves as an effective mechanism to mitigate biases that commonly arise from reverse causation and confounding (22, 23). Furthermore, MR analysis utilizes single nucleotide polymorphisms (SNPs) as the primary exposure factors for investigating their causal associations with outcomes. These SNPs strictly adhere to the tenets of Mendelian genetics, signifying that allele genes are haphazardly assigned to descendant gametes during gamete formation, endowing them with characteristics akin to those in a RCT (22, 24). Thus, MR introduces an innovative avenue for elucidating the causal relationship between migraines and AD.

In this research, we employed SNPs from Genome-Wide Association Studies (GWAS) as instrumental variables (IVs). Utilizing Univariable MR (UVMR), we investigated the causal relationship between migraines and AD. Subsequently, we constructed models incorporating migraines and various risk factors associated with AD. Employing multivariate MR (MVMR), we explored the direct impact of migraines on the risk of AD occurrence, considering the presence of AD-related risk factors.

## Methods

### Study design

In this study, we chose migraine as the independent variable and AD as the outcome, employing the UVMR method to investigate the causal effect of migraine on the risk of AD occurrence. To ensure the reliability of our findings, we utilized two independent datasets related to AD, conducting separate UVMR analyses on each dataset. The results from these analyses were meticulously synthesized using meta-analysis, thereby enhancing the robustness of our research conclusions.

**Figure 1:**
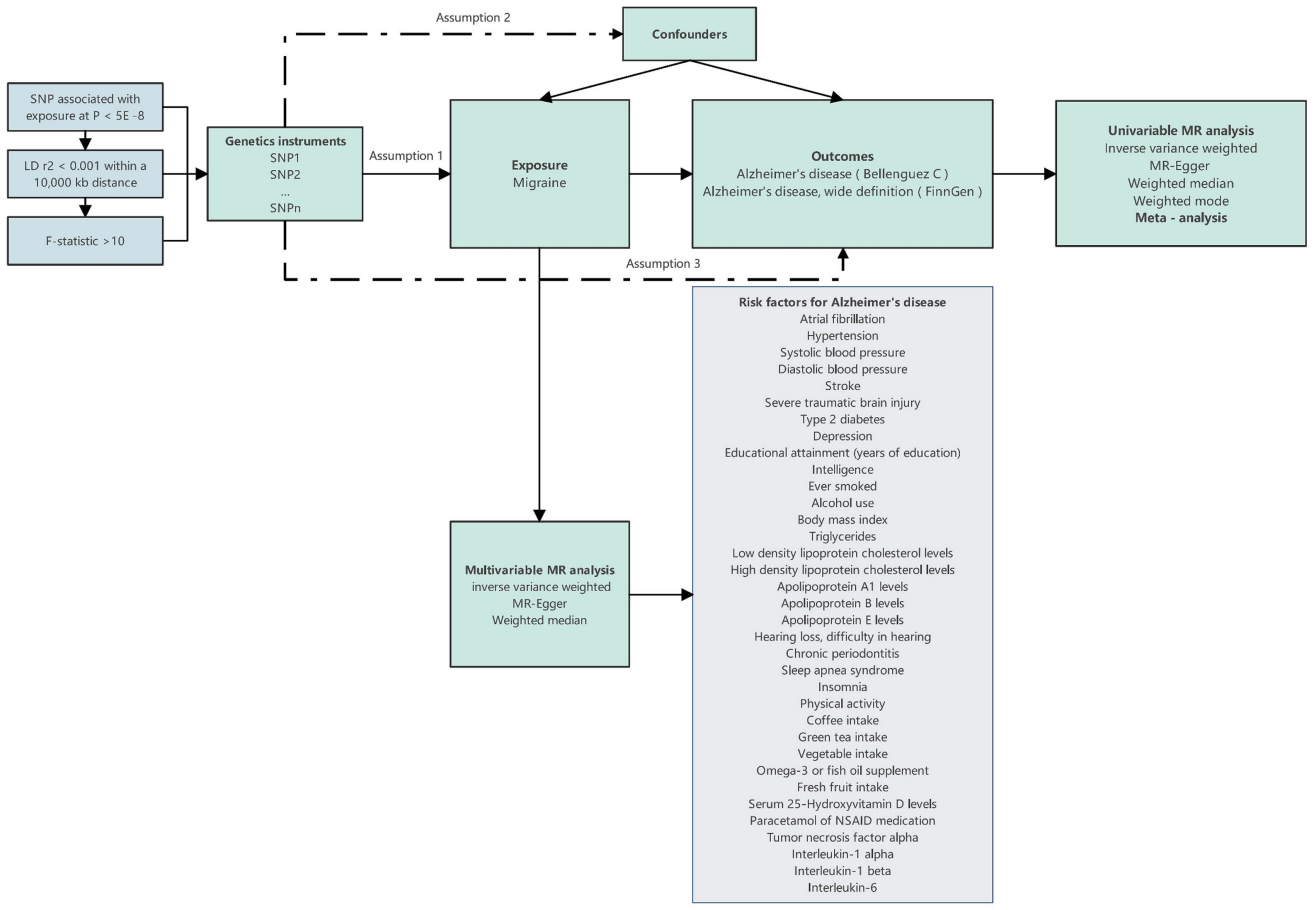
Design overview and instrumental variable assumptions in this Mendelian randomization study. The essential prerequisites for genetic variation to fulfill the instrumental variables criteria in this study are as follows: Assumption 1: The genetic variant must exhibit an association with the exposure; Assumption 2: it must not exhibit a relationship with any confounding factors that could affect the connection between the exposure and outcome; and Assumption 3: the genetic variant should not directly influence the outcome, except through its association with the exposure.

Subsequently, we introduced the MVMR method to account for the presence of multiple potential risk factors, examining the independent impact of migraine on the onset risk of AD. In this process, migraine was considered alongside various potential risk factors associated with AD, forming a comprehensive model. Within this model, multifactorial elements were treated as exposure variables, with AD serving as the study’s outcome. Through the application of this multifactorial model, we were able to comprehensively and accurately assess the independent influence of migraine on the risk of AD occurrence. The specific workflow of the entire research process can be referred to in Figure 1.

### Data sources

The data regarding migraine were derived from the study conducted by Dönertaş et al. (25), encompassing 13,971 patients and 470,627 controls, with a total of 9,587,836 SNPs analyzed. As for AD, data were sourced from the research studies led by Bellenguez et al. (26) (Cases: 39,106, Control: 46,828, SNPs: 20,921,626) and the FinnGen database (Cases: 5,918, Control: 111,471, SNPs: 16,379,561). The risk factors for AD included in the MVMR were summarized from multiple reviews (3, 4, 27). These factors encompass Atrial fibrillation (AF), Hypertension, Systolic blood pressure (SBP), Diastolic blood pressure (DBP), Stroke, Severe traumatic brain injury (STBI), Type 2 diabetes, Depression, Educational attainment (years of education), Intelligence, Ever smoked, Alcohol use, Body mass index (BMI), Triglycerides, Low-density lipoprotein cholesterol levels (LDL), High-density lipoprotein cholesterol levels (HDL), Apolipoprotein A1 levels, Apolipoprotein B levels, Apolipoprotein E levels, Hearing loss, Chronic periodontitis, Sleep apnea syndrome, Insomnia, Physical activity, Filtered coffee intake, Green tea intake, Cooked vegetable intake, Omega-3 or fish oil supplement, Fresh fruit intake, Serum 25-Hydroxyvitamin D levels, Paracetamol or NSAID medication, Tumor necrosis factor alpha (TNFα), Interleukin (IL)-1α, IL-1β, and IL-6, totaling 35 risk factors. Data pertaining to these factors were obtained from 10 distinct studies (25, 28–36) and four databases (FinnGen, Within family GWAS consortium, MRC-IEU, and Neale lab).

All populations included in the datasets are of European ancestry origin. Comprehensive details of the datasets included in the study are available in Supplementary Table 1.

### Selection of IVs

In this investigation, SNPs were employed as IVs to estimate the causal impact of the exposure on the outcome. SNPs, randomly allocated during meiosis, are largely immune to traditional biases inherent in observational studies, such as confounding, reverse causality, and measurement errors. They prove invaluable for exploring causal relationships with outcomes, provided certain assumptions are met.

The essential prerequisites for genetic variation to fulfill the IVs criteria in this study are as follows: (1) The genetic variant must exhibit an association with the exposure; (2) it must not exhibit a relationship with any confounding factors that could affect the connection between the exposure and outcome; and (3) the genetic variant should not directly influence the outcome, except through its association with the exposure.

The IVs selection process adhered to stringent criteria to ensure their validity and reliability. IVs were selected based on their significant genome-wide associations with the exposure (*p* < 5e-8), a minor allele frequency above 0.01 in the outcome, and low linkage disequilibrium r2 within a 10,000 kb distance.

SNPs associated with confounding factors or outcomes according to the Phenoscanner database^1^ were excluded from the study. The proportion of variance explained by individual SNPs was calculated using the formula $R^{2}=\frac{2\times \beta^{2}\times EAF\times \left(1-EAF\right)}{2\times \beta^{2}\times EAF\times \left(1-EAF\right)+2\times SE^{2}\times N\times EAF\times \left(1-EAF\right)}\text{.}$ In MR analysis, R^2^ represents the “proportion of variance explained,” quantifying the strength of the relationship between genetic variation (used as IVs) and a specific outcome variable. β^2^ indicates the regression coefficient, indicating the strength of the association between the IVs and the outcome. EAF, or effect allele frequency, represents the frequency of genetic variation. SE^2^ represents the variance of the outcome variable, indicating the extent of variability in the outcome variable. It serves as a statistical measure of the outcome variable’s distribution. N represents the number of individuals included in the study.

To assess the robustness of the IVs, we computed the F-statistic using the formula $F=\frac{\left(N-k-1\right)}{k}\times \frac{R^{2}}{\left(1-R^{2}\right)}$ , where N represents the number of samples exposed to the GWAS, k is the number of IVs, and R^2^ indicates the proportion of exposure explained by the IVs. The F-statistic values were employed to evaluate the IVs, with instruments having low values (less than 10) considered weak, potentially introducing biases into the results. Consequently, the study’s conclusions were approached with caution, recognizing the limitations associated with weak IVs.

### Statistical analysis

The beta values of SNPs in the exposure dataset will undergo Z-score normalization. In the context of UVMR, our primary analytical approach centered on the utilization of the inverse variance weighted (IVW) method. This method is particularly valuable for yielding precise causal estimates, operating under the fundamental assumption of the validity of IVs (37). In instances characterized by heterogeneity among the selected IVs, we employed a random-effects IVW method to account for this variability. Conversely, in scenarios where no significant heterogeneity was observed, a fixed-effects model was deemed appropriate for our analysis.

To enhance the robustness of our analysis, we employed Supplementary methods including MR Egger (38), weighted median (WM) (39), and weighted mode (40) for sensitivity analyses. In order to gauge heterogeneity, we utilized Cochran’s Q statistic in conjunction with the MR-Egger methods (38, 41), where a *p*-value exceeding 0.05 indicated the absence of significant heterogeneity within the dataset.

To investigate potential pleiotropy, we conducted MR-Egger intercept and MR-PRESSO tests (42). A *p*-value exceeding 0.05 from these tests provided strong evidence suggesting the absence of pleiotropic effects among the IVs. Additionally, a “leave-one-out (LOO)” analysis was conducted, allowing us to meticulously assess the influence of individual SNPs on the causal relationship between the exposure and the outcome, thereby ensuring a comprehensive evaluation of our findings.

In our rigorous evaluation of the directional causative relationship between exposures and outcomes, we conducted the MR Steiger test (43). A *p*-value below 0.05 in this test indicates the correctness of the causal direction. To address the challenge of multiple testing, we utilized a Bonferroni correction threshold of *p* < 0.025 (0.05/2, considering the two outcome traits associated with AD). *p*-values falling between 0.025 and 0.05 were deemed suggestive indicators of potential causality, warranting further validation. Associations were considered statistically significant when p-values were below 0.025 in the IVW method, and when the results from the MR-Egger, WM, and weighted mode methods aligned with those of IVW.

Our assessment of the causal relationship between Migraine and the risk of AD was determined through a meta-analysis. This meta-analysis was conducted employing a fixed-effects IVW model using Review Manager 5.4 software, ensuring a robust and comprehensive evaluation of the findings.

In the MVMR, we selected the larger dataset pertaining to AD (26) for our analysis. The IVW method served as our primary analytical tool. Additionally, we incorporated MR-Egger and MR-WM as supplementary approaches. The horizontal pleiotropy of IVs was evaluated through MR-Egger, rejecting the presence of horizontal pleiotropy when the *p*-value exceeded 0.05.

To identify heterogeneity, we employed the IVW heterogeneity Q test. The null hypothesis of this test assumed uniform effect sizes across all genes. If the *p*-value in the IVW heterogeneity Q test was below 0.05, indicating the presence of heterogeneity, alternative methods such as WM or MR-Egger were considered. These methods offered more reliable causal estimates in the presence of heterogeneity. MR-Egger regression enabled estimation while considering horizontal pleiotropy, albeit with slightly reduced precision (38). If the *p*-value from MR-IVW’s Q test was less than 0.05, we deemed MR-Egger results as supportive when the effect estimate aligned with MR-IVW, and the MR-Egger Q test was statistically nonsignificant (*p* > 0.05). If both Q tests yielded *p*-values below 0.05, we relied on MR-WM results, an additional measure ensuring accurate causality estimation if at least 50% of the analysis’s weight originated from valid instrumental variables (39). The reliability of the outcomes was affirmed only when all three methods produced consistent results’ direction.

The effect estimates were meticulously presented as odds ratios (OR), each accompanied by its corresponding 95% confidence intervals (CI). All MR analyses were diligently executed employing R software (version 4.2.2) and a selection of indispensable R Packages including “TwoSampleMR,” “MendelianRandomization,” “psych,” and “MRPRESSO.” For the purpose of data visualization, we effectively harnessed the capabilities of the R Package “ggplot2 [3.3.6].”

## Results

### UVMR analyses

Following the predetermined criteria for SNP selection, 12 SNPs were extracted after harmonizing the exposure and outcome. Subsequent scrutiny via the Phenoscanner database revealed the association of rs9349379 with SBP and various cardiovascular diseases (Supplementary Table 2). Consequently, this specific SNP was excluded from the analysis, resulting in a final set of 11 SNPs utilized as IVs for the MR analysis (Supplementary Table 3). Notably, all these SNPs exhibited *F*-values exceeding 10, mitigating the potential influence of weak IVs (Supplementary Table 3).

**Figure 2:**
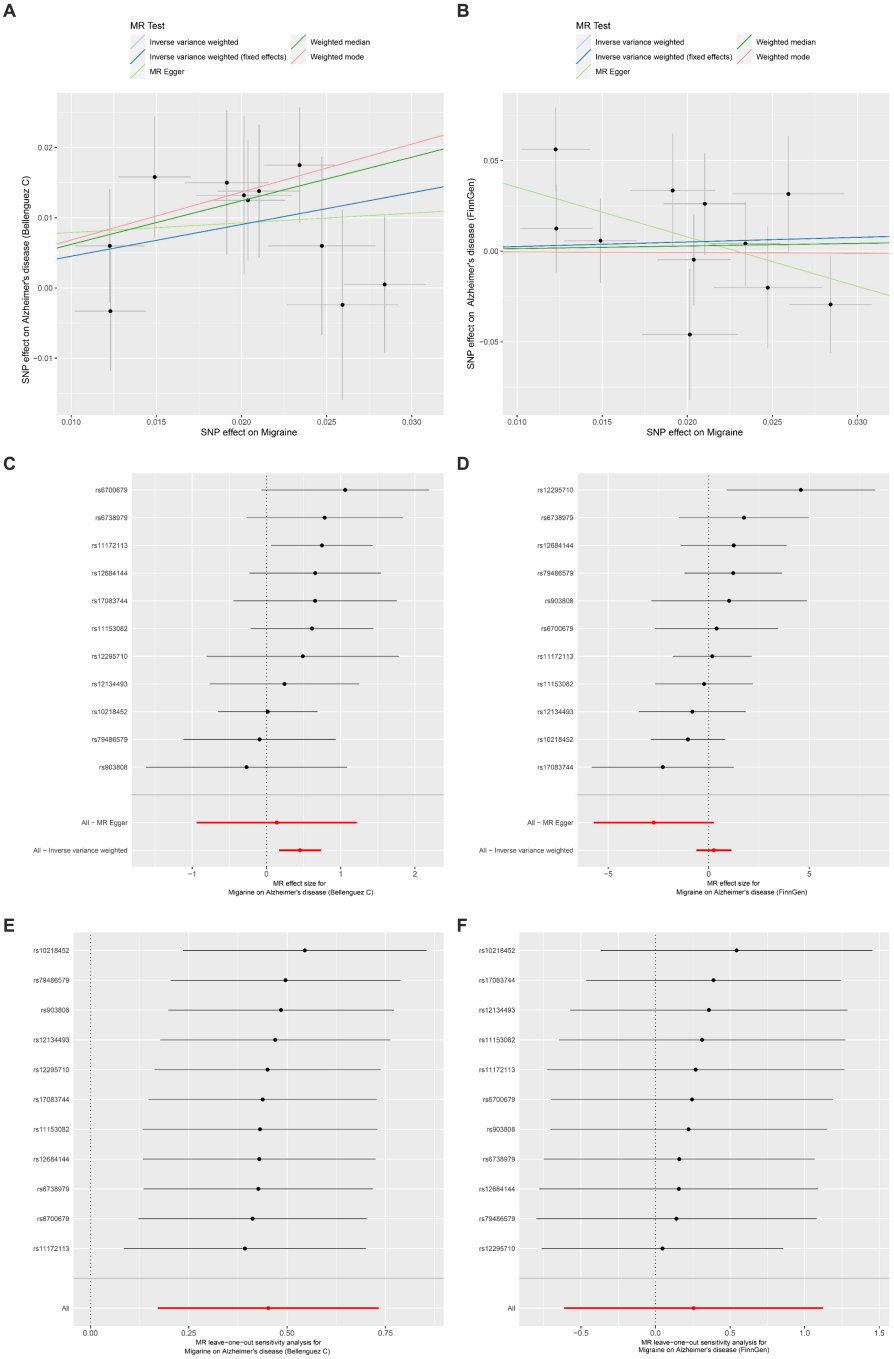
Univariable Mendelian randomization analyses. **(A)** Individual MR estimates of SNP effects on Migraine and Alzheimer’s disease risk from Bellenguez C et al. displayed in a scatter plot. **(B)** Scatter plot showing individual MR estimates of SNP effects on Migraine and Alzheimer’s disease risk from FinnGen. **(C)** Forest plot illustrating the potential causal association between Migraine and Alzheimer’s disease risk based on data from Bellenguez C et al. **(D)** Forest plot presenting the potential causal association between Migraine and Alzheimer’s disease risk using data from FinnGen. **(E)** Leave-one-out sensitivity analysis demonstrating the MR analysis for Migraine on Alzheimer’s disease risk from Bellenguez C et al., indicating the impact of excluding individual SNPs. **(F)** Leave-one-out sensitivity analysis illustrating the MR analysis for Migraine on Alzheimer’s disease risk from FinnGen, highlighting the effect of excluding individual SNPs.

The outcomes of the MR analysis are detailed in Table 1 and Figure 2. The absence of heterogeneity, as indicated in Table 2, allowed for the application of the fixed-effects IVW method. In the dataset presented by Bellenguez et al. (26), gene prediction for migraine demonstrated a causal effect on the elevated risk of AD (OR [95%CI]: 1.5717 [1.1868–2.0814], *p* = 0.0016) (Table 1; Figures 2A,C). In contrast, the FinnGen dataset did not exhibit a significant association between the two conditions (OR [95% CI]: 1.2904 [0.5419–3.0730], *p* = 0.5646) (Table 1; Figures 2B,D).

**Table 1:** Univariable Mendelian randomization analyses of migraine on the risk of Alzheimer’s disease.

Exprosure	Outcome	Methods	nSNPs	b	SE	pval	OR	or_lci95	or_uci95
Migraine	Alzheimer’s disease (Bellenguez C)	IVW	11	4.52E−01	1.43E−01	1.60E−03	1.57E+00	1.19E+00	2.08E+00
		MR Egger	11	1.38E−01	5.50E−01	8.07E−01	1.15E+00	3.90E−01	3.38E+00
		Weighted median	11	6.21E−01	1.91E−01	1.14E−03	1.86E+00	1.28E+00	2.70E+00
		Weighted mode	11	6.83E−01	3.00E−01	4.62E−02	1.98E+00	1.10E+00	3.57E+00
	Alzheimer’s disease (FinnGen)	IVW	11	2.55E−01	4.03E−01	5.27E−01	1.29E+00	5.85E−01	2.85E+00
		MR Egger	11	−2.73E+00	1.52E+00	1.06E−01	6.49E−02	3.29E−03	1.28E+00
		Weighted median	11	1.43E−01	5.56E−01	7.96E−01	1.15E+ 00	3.88E−01	3.43E+00
		Weighted mode	11	−3.58E−02	7.79E−01	9.64E−01	9.65E−01	2.10E−01	4.44E+00

SE, standard error; OR, odds ratio; CI, confidence intervals; SNP, Single nucleotide polymorphism.

**Table 2:** Sensitivity analysis and Steiger test in Univariable Mendelian randomization analyses.

Exprosure	Outcome	Heterogeneity test	Pleiotropy test	Pleiotropy test	Outlier	Steiger test *p*
		MR-Egger *p*	MR-Egger *p*	PRESSO *p*		
Migraine	Alzheimer’s disease (Bellenguez C)	7.11E−01	5.69E−01	7.51E−01	NA	2.67E−18
	Alzheimer’s disease (FinnGen)	5.45E−01	7.21E−02	3.31E−01	NA	3.12E−20

**Figure 3:**
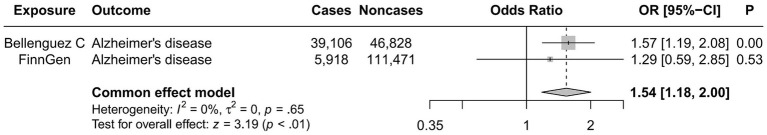
Meta-analysis the results of univariable Mendelian randomization analyses.

Upon conducting a meta-analysis of data from Bellenguez et al. (26) and the FinnGen consortium, the combined OR [95% CI] was determined to be 1.54 [1.18, 2.00] with a *p*-value less than 0.01. This finding further solidified the causal effect of migraine in increasing the susceptibility to AD, as depicted in Figure 3.

The MR-Egger intercept test and MR-PRESSO test did not reveal any horizontal pleiotropy (Table 2). Additionally, LOO analysis demonstrated that no individual IV exerted a disproportionate influence on the causal effect (Figures 2E,F). Moreover, the Steiger analysis indicated that the selected SNPs explained a higher proportion of variance in the exposure variable compared to the outcome. This result solidified the correct directionality of the causal effect from exposure to outcome and ruled out the possibility of reverse causation (Table 2).

### MVMR analyses

In the MVMR analysis, the IVs utilized were sourced from SNPs in both the Migraine and risk factors’ datasets (Supplementary Table 4). We utilized the MR-IVW method for adjusting these risk factors, given the absence of heterogeneity when Adjusting for all risk factors. Importantly, no horizontal pleiotropy was observed in any of the adjustments made, as evidenced in Supplementary Table 5.

After adjusting for specific factors, including AF (OR [95% CI]: 1.7571 [1.2941–2.3856], *p* = 0.0003), Hypertension (OR [95% CI]: 1.7928 [1.2624–2.5461], *p* = 0.0011), SBP (OR [95% CI]: 1.5528 [1.1228–2.1474], *p* = 0.0078), Stroke (OR [95% CI]: 1.5991 [1.2041–2.1235], *p* = 0.0012), STBI (OR [95% CI]: 1.5222 [1.1407–2.0314], *p* = 0.0043), Type 2 diabetes (OR [95% CI]: 1.5444 [1.1455–2.0821], *p* = 0.0044), Depression (OR [95% CI]: 1.4737 [1.1049–1.9656], *p* = 0.0083), Educational attainment (years of education) (OR [95% CI]: 1.7749 [1.1780–2.6742], *p* = 0.0061), Intelligence (OR [95% CI]: 1.6501[1.2041–2.2613], *p* = 0.0018), Ever smoked (OR [95% CI]: 1.5992 [1.1950–2.1400], *p* = 0.0016), Alcohol use (OR [95% CI]: 1.6847 [1.2316–2.3046], p = 0.0011), Triglycerides (OR [95% CI]: 1.6451 [1.2149–2.2276], *p* = 0.0013), LDL (OR [95% CI]: 1.7063 [1.1602–2.5094], *p* = 0.0066), HDL (OR [95% CI]: 1.5356 [1.0827–2.1779], *p* = 0.0161), Apolipoprotein A1 levels (OR [95% CI]: 1.4849 [1.0966–2.0107], *p* = 0.0106), Apolipoprotein B levels (OR [95% CI]: 1.8388 [1.2888–2.6235], *p* = 0.0008), Apolipoprotein E levels (OR [95% CI]: 1.5715 [1.1776–2.0972], *p* = 0.0021), Hearing loss, difficulty in hearing (OR [95% CI]: 1.5770 [1.1907–2.0888], *p* = 0.0015), Chronic periodontitis (OR [95% CI]: 1.5488 [1.1321–2.1190], *p* = 0.0062), Sleep apnea syndrome (OR [95% CI]: 1.5442 [1.1648–2.0472], *p* = 0.0025), Insomnia (OR [95% CI]: 1.5506 [1.1691–2.0566], *p* = 0.0023), Physical activity (OR [95% CI]: 1.4996 [1.1059–2.0335], *p* = 0.0091), Coffee intake (OR [95% CI]: 1.5389 [1.1298–2.0960], *p* = 0.0063), Green tea intake (OR [95% CI]: 1.7197 [1.2463–2.3729], *p* = 0.0010), Vegetable intake (OR [95% CI]: 1.6288 [1.2157–2.1822], *p* = 0.0011), Omega-3 or fish oil supplement (OR [95% CI]: 1.4892 [1.0902–2.0343], *p* = 0.0123), Fresh fruit intake (OR [95% CI]: 1.6459 [1.2250–2.2113], *p* = 0.0009), Serum 25-Hydroxyvitamin D levels (OR [95% CI]: 1.6132 [1.1118–2.3408], *p* = 0.0118), Paracetamol or NSAID medication (OR [95% CI]: 1.4663[1.0539–2.0400], *p* = 0.0231), IL-1α (OR [95% CI]: 1.5711 [1.1863–2.0808], *p* = 0.0016), IL-1β (OR [95% CI]: 1.5169 [1.1405–2.0174], *p* = 0.0042), and IL-6 (OR [95% CI]: 1.5414 [1.1487–2.0683], *p* = 0.0039), the relationship between Migraine and an increased risk of AD persisted (Table 3; Supplementary Table 6). The observed association remained consistently directional across various analytical methods, including IVW, MR-Egger, and WM approaches (Supplementary Table 6).

**Table 3:** Multivariable Mendelian randomization analyses.

Adjusted for	Methods	nSNPs	beta	SE	*p*-value	or	or_lci95	or_uci95
Atrial fibrillation	IVW	11	0.5636	0.1560	0.0003	1.7571	1.2941	2.3856
Hypertension	IVW	7	0.5838	0.1790	0.0011	1.7928	1.2624	2.5461
Systolic blood pressure	IVW	7	0.4400	0.1654	0.0078	1.5528	1.1228	2.1474
Diastolic blood pressure	IVW	6	0.3450	0.2597	0.1840	1.4120	0.8487	2.3493
Stroke	IVW	11	0.4694	0.1447	0.0012	1.5991	1.2041	2.1235
Severe traumatic brain injury	IVW	11	0.4202	0.1472	0.0043	1.5222	1.1407	2.0314
Type 2 diabetes	IVW	9	0.4346	0.1524	0.0044	1.5444	1.1455	2.0821
Depression	IVW	11	0.3877	0.1470	0.0083	1.4737	1.1049	1.9656
Educational attainment (years of education)	IVW	8	0.5737	0.2091	0.0061	1.7749	1.1780	2.6742
Intelligence	IVW	9	0.5008	0.1608	0.0018	1.6501	1.2041	2.2613
Ever smoked	IVW	11	0.4695	0.1486	0.0016	1.5992	1.1950	2.1400
Alcohol use	IVW	10	0.5216	0.1599	0.0011	1.6847	1.2316	2.3046
Body mass index	IVW	4	0.3241	0.2410	0.1788	1.3827	0.8621	2.2176
Triglycerides	IVW	10	0.4978	0.1547	0.0013	1.6451	1.2149	2.2276
Low density lipoprotein cholesterol levels	IVW	10	0.5343	0.1968	0.0066	1.7063	1.1602	2.5094
High density lipoprotein cholesterol levels	IVW	11	0.4289	0.1783	0.0161	1.5356	1.0827	2.1779
Apolipoprotein A1 levels	IVW	10	0.3953	0.1547	0.0106	1.4849	1.0966	2.0107
Apolipoprotein B levels	IVW	6	0.6091	0.1813	0.0008	1.8388	1.2888	2.6235
Apolipoprotein E levels	IVW	11	0.4520	0.1472	0.0021	1.5715	1.1776	2.0972
Hearing loss	IVW	11	0.4556	0.1434	0.0015	1.5770	1.1907	2.0888
Chronic periodontitis	IVW	11	0.4375	0.1599	0.0062	1.5488	1.1321	2.1190
Sleep apnea syndrome	IVW	11	0.4345	0.1439	0.0025	1.5442	1.1648	2.0472
Insomnia	IVW	11	0.4386	0.1441	0.0023	1.5506	1.1691	2.0566
Physical activity	IVW	10	0.4052	0.1554	0.0091	1.4996	1.1059	2.0335
Coffee intake	IVW	11	0.4310	0.1577	0.0063	1.5389	1.1298	2.0960
Green tea intake	IVW	10	0.5421	0.1643	0.0010	1.7197	1.2463	2.3729
Vegetable intake	IVW	10	0.4878	0.1492	0.0011	1.6288	1.2157	2.1822
Omega-3 or fish oil supplement	IVW	10	0.3983	0.1591	0.0123	1.4892	1.0902	2.0343
Fresh fruit intake	IVW	10	0.4983	0.1507	0.0009	1.6459	1.2250	2.2113
Serum 25-Hydroxyvitamin D levels	IVW	6	0.4782	0.1899	0.0118	1.6132	1.1118	2.3408
Paracetamol of NSAID medication	IVW	11	0.3827	0.1685	0.0231	1.4663	1.0539	2.0400
Tumor necrosis factor alpha	IVW	11	0.2160	0.2021	0.2852	1.2411	0.8352	1.8443
Interleukin-1 alpha	IVW	11	0.4518	0.1434	0.0016	1.5711	1.1863	2.0808
Interleukin-1 beta	IVW	11	0.4166	0.1455	0.0042	1.5169	1.1405	2.0174
Interleukin-6	IVW	11	0.4327	0.1500	0.0039	1.5414	1.1487	2.0683

SE, standard error; OR, odds ratio; CI, confidence intervals; IVW, inverse variance weighted.

However, after adjusting for DBP (OR [95% CI]: 1.4120 [0.8487–2.3493], *p* = 0.1840) and TNFα (OR [95% CI]: 1.2411 [0.8352–1.8443], *p* = 0.2852), no discernible association was detected between the genetic predisposition to migraine and the risk of AD (Table 3; Supplementary Table 6). Upon adjusting for BMI, the IVW method revealed no substantial link between migraine and AD risk (OR [95% CI]:1.3827 [0.8621–2.2176], *p* = 0.1787). However, it is noteworthy that the direction of this association, as indicated by IVW, diverged from the outcomes derived from the MR-Egger methods. Consequently, this particular finding lacks robustness and stability, warranting careful interpretation (Supplementary Table 6).

## Discussion

Migraine and AD both represent severe neurological disorders, imposing significant burdens on patients and society alike. In this study, a comprehensive exploration of the causal relationship between migraine and the risk of developing AD was conducted, employing a methodological approach combining UVMR with Meta-analysis. Subsequently, MVMR was employed to delve into the direct causal effects of migraine on AD within the context of multiple coexisting risk factors for AD. The findings from our study, synthesized through Meta-analysis, unequivocally established a notable causal link between the genetic predisposition to migraine and an increased risk of AD. However, upon adjusting for variables such as DBP and TNFα, the previously observed association between the genetic predisposition to migraine and AD risk dissipated. Our subsequent sensitivity analyses further bolstered the reliability and stability of our research results. To the best of our knowledge, this study marks a pioneering effort, being the first to incorporate such a myriad of risk factors in the realm of MVMR, aiming to thoroughly investigate the intricate relationship between migraine and the risk of developing AD.

The relationship between migraine and AD has been a subject of extensive interest. Early studies did not reveal a clear connection between migraine and AD (17, 20, 44–46). In fact, one meta-analysis even indicated a significant negative correlation between the two conditions (47). However, these studies often failed to simultaneously consider various common AD risk factors such as hyperlipidemia, depression, diabetes, and stroke (15). Moreover, negative results could be attributed to disparities in the diagnostic criteria for cognitive impairment, methodological strategies, and differences in the sizes of the study populations (16). Recent large-scale national retrospective studies have shed new light on the relationship between migraine and increased AD risk. Two studies from South Korea, analyzing data from the 2002–2019 Korean National Health Insurance Health Screening Cohort, found a heightened risk of AD dementia in individuals with a history of migraine (13, 14). Although these studies primarily focused on Asian populations, caution is necessary when generalizing the findings to other ethnic groups. Conversely, research conducted by Karel Kostev et al. (15) in the United Kingdom, involving a retrospective analysis of 7,454 patients diagnosed with migraine from January 1997 to December 2016 in 67 general practitioner clinics, revealed a positive correlation between migraine diagnosis and AD. Another study surveyed 679 community-dwelling residents aged 65 and above in Canada, adjusting for confounding factors such as age, gender, education, and depression, as well as intervening variables like hypertension, myocardial infarction, other heart diseases, stroke, and diabetes. It identified migraine as a significant risk factor for AD (16). Considering our dataset’s European origins, we meticulously chose two vastly different datasets for our analysis and synthesized the results through meta-analysis to enhance the reliability of our findings. Our comprehensive analysis unequivocally demonstrated a significant causal effect of migraine in increasing the risk of AD, aligning with the outcomes of the aforementioned studies.

Given the significant difference in the age of onset between migraine and AD, ensuring a longitudinal relationship between the two diseases necessitates a study with a sufficiently long follow-up period. Separating causal factors between them from confounding variables over such an extended follow-up period poses a highly challenging task, especially without employing the method we utilized, involving genetic variations as IVs in our study. Effect estimates derived from MR studies are generally considered as “lifetime effects” since the lineage-specific genetic variations utilized in this method are fixed from pregnancy onwards (48). Furthermore, a study revealed that patients diagnosed with migraine for less than 5 years exhibited a notably stronger association with dementia compared to those diagnosed with migraine for a longer duration. This finding raised the possibility of a potential reverse causal relationship between migraine and dementia. However, the study also observed a significant increase in the risk of developing dementia among patients diagnosed with migraine, especially those diagnosed before the age of 60, providing support for the longitudinal relationship between the two conditions (14). Through Steiger’s test, we confirmed that the IVs we employed showed a significantly stronger correlation with migraine than with AD (43). This confirmation establishes the directionality of the longitudinal relationship from migraine to AD, thereby eliminating the possibility of a reverse causal effect.

The onset of AD is intricately associated with multiple risk factors that span across the entire lifespan (49, 50). Intriguingly, some of these risk factors are also linked to migraine (51–54). Moreover, migraine may even trigger the occurrence of some of these risk factors (55). Utilizing MVMR analysis, we were able to control these confounding factors, enabling an exploration of the direct causal effect of migraine on AD, thereby enhancing the credibility of the causal relationship between the two. Previous studies have indicated that factors such as STBI, Depression, Ever smoked, Alcohol use, Hearing loss, Chronic periodontitis, Sleep apnea syndrome, Insomnia, Physical activity, Coffee intake, Green tea intake, Vegetable intake, Omega-3 or fish oil supplement, Fresh fruit intake, Serum 25-Hydroxyvitamin D levels, Paracetamol or NSAID medication are all correlated with the risk of developing AD (4, 27, 56–59). According to our research findings, migraine significantly increases the risk of developing AD independently of these aforementioned risk factors. This implies that these diseases, lifestyles, dietary habits, and medication use have a relatively minor causal effect on the increased risk of AD due to migraine.

Furthermore, intelligence and educational attainment had no impact on the relationship between the two, indicating that regardless of educational attainment or intelligence level, individuals with migraines should remain vigilant for subsequent development of AD.

Through MVMR analysis, our study unveiled intriguing findings. For instance, there is a significant correlation between cardiovascular diseases and the increased risk of AD (4). Our research indicated that even when accounting for factors such as AF, Hypertension, SBP, Stroke, Type 2 diabetes, Triglycerides, LDL, HDL, apolipoprotein A1 levels, apolipoprotein B levels, and apolipoprotein E levels, migraine continues to elevate the risk of AD. This observation aligns with prior research outcomes. In a substantial retrospective study conducted by Kim and colleagues, it was discovered that after adjusting for AD risk factors such as diabetes, hypertension, and lipid abnormalities, migraine still exhibits a notable impact on AD development (13).

However, our results demonstrated that the association between migraine and AD risk could be eliminated by considering DBP. This suggests that specific physiological indicators, rather than an overall diseased or unhealthy state, mediate the relationship between migraine and the risk of developing AD. Additionally, similar phenomena were observed in the context of neuroinflammation. The immune response of the nervous system significantly contributes to the pathophysiological processes of AD (60–62). Simultaneously, there is a close association between neuroinflammation and migraine (63–65). Several cytokines, including TNFα, IL-1, and IL-6, are implicated in the pathogenesis of migraine (66, 67). Our results indicated that the effect of migraine in increasing the risk of AD development might be mediated by TNFα, while IL-1α, IL-1β, and IL-6 do not play a role in this process. This discovery not only reveals the specific role of certain inflammatory factors in the interplay between migraine and AD but also provides new insights for the development of preventive and therapeutic medications tailored for specific high-risk groups of AD.

Determining the causal effect of migraine on the risk of AD holds significant clinical importance. This determination allows for the early identification of individuals at high risk, underscoring the recommendation for regular cognitive decline and dementia screening among those with migraines. Additionally, providing appropriate treatment and management for migraine sufferers is crucial, as it may aid in preventing subsequent dementia development. By screening migraine patients early for cognitive decline and actively addressing potential intervention factors, we can slow the progression of dementia, thereby enhancing patients’ quality of life (68). Furthermore, the causal effect of migraine on AD implies that these two conditions may share common underlying mechanisms. This finding contributes to a better understanding of the pathogenesis of both diseases and provides insights for future research directions. Drawing from existing effective migraine treatment methods, we can leverage this knowledge to develop new AD prevention strategies and treatments targeting migraine and related intervention factors. This endeavor can improve patient health outcomes and pave the way for novel approaches to AD prevention and management.

Our study carries several limitations that merit consideration. Firstly, our migraine dataset did not support further stratified analyses, such as distinguishing between chronic migraine, female patients, or younger individuals. These factors may exert an influence on the outcomes. Research has indicated that patients with chronic migraines exhibit a higher rate of AD development compared to those with episodic migraines, and young migraine sufferers also present a greater risk of AD onset when compared to the control group (13). Multiple studies have further suggested that there is a significant positive correlation between migraine and AD in females, whereas this association is not as pronounced in males (15, 16, 69, 70). Moreover, the frequency and severity of migraine attacks may also impact the interrelation between these two conditions, demanding a more precise analysis. Secondly, although our study adjusted for 35 risk factors associated with AD, given the multitude of factors influencing AD risk, there might still be other elements affecting the results. Future research is needed to delve deeper into this matter. Lastly, it’s worth noting that our data was derived from European ancestral populations. While our findings received support from large-scale retrospective studies involving Asian populations (13, 14), it is essential to validate the generalizability of our results to other populations through the analysis of datasets from local populations.

## Conclusion

This study employed MR analysis to unveil a substantial association between migraine and the risk of AD. Remarkably, this association remains robust even after meticulous adjustments for various potential confounding factors. Nevertheless, with specific factors, including DBP and TNFα considered, this association dissipates, indicating the intricate interplay of diverse factors. This research not only enriches our comprehension of the migraine-AD relationship but also imparts valuable insights for clinical practice and disease.

## Data availability statement

All data supporting the findings of this study are provided within the paper and its Supplementary material. All additional information will be made available upon reasonable request from the corresponding author.

## Ethics statement

Ethical approval was not required for the study involving humans in accordance with the local legislation and institutional requirements. Written informed consent to participate in this study was not required from the participants or the participants’ legal guardians/next of kin in accordance with the national legislation and the institutional requirements.

## Author contributions

CX: Data curation, Formal analysis, Investigation, Validation, Writing – original draft. WW: Data curation, Formal analysis, Investigation, Validation, Writing – original draft. YF: Conceptualization, Methodology, Project administration, Resources, Software, Supervision, Visualization, Writing – original draft, Writing – review & editing. SZ: Conceptualization, Project administration, Supervision, Writing – review & editing.

## Footnotes

^1^ http://www.phenoscanner.medschl.cam.ac.uk/

## Glossary

AD: Alzheimer’s Disease
UVMR: Univariable Mendelian Randomization
MVMR: Multivariable Mendelian Randomization
SNPs: Single Nucleotide Polymorphisms
RCTs: Randomized Controlled Trials
GWAS: Genome-Wide Association Studies
IVs: Instrumental Variables
AF: Atrial Fibrillation
SBP: Systolic Blood Pressure
DBP: Diastolic Blood Pressure
STBI: Severe Traumatic Brain Injury
BMI: Body Mass Index
LDL: Low-Density Lipoprotein Cholesterol Levels
HDL: High-Density Lipoprotein Cholesterol Levels
TNFα: Tumor Necrosis Factor Alpha
IL: Interleukin
EAF: Effect Allele Frequency
IVW: Inverse Variance Weighted
WM: Weighted Median
LOO: Leave-One-Out
OR: Odds Ratios
CI: Confidence Intervals
